# Grating-Based Fiber-Optic Sensing Using a Single Packaged FBG for Boundary-Dependent Motor Vibration-State Transitions

**DOI:** 10.3390/s26133994

**Published:** 2026-06-24

**Authors:** Cheng-Yu Lin, Pei-Chung Liu, Cheng-Kai Yao, Shao-Chi Huang, Shi-Jia Huang, Sheng-Jie Chen, Peng-Chun Peng

**Affiliations:** Department of Electro-Optical Engineering, National Taipei University of Technology, Taipei 10608, Taiwan; t114659005@ntut.edu.tw (C.-Y.L.); t113658043@ntut.edu.tw (P.-C.L.); t111659004@ntut.org.tw (C.-K.Y.); t114658072@ntut.org.tw (S.-C.H.); t113658085@ntut.edu.tw (S.-J.H.); t113658078@ntut.edu.tw (S.-J.C.)

**Keywords:** fiber Bragg grating, motor vibration-state monitoring, boundary-dependent vibration, adaptive variational mode decomposition, autoencoder alarm indication

## Abstract

This study demonstrates single-channel fiber Bragg grating (FBG) sensing for relative vibration-state monitoring of a motor–support system under angle-dependent boundary conditions. A packaged FBG accelerometer-type sensing unit was mounted on the motor–support structure, and the reflected Bragg wavelength was recorded as a one-dimensional optical vibration response. Because the sensor was installed away from the rotating shaft, the measured wavelength fluctuation was interpreted as a coupled vibration-sensitive response of the motor, fixture, sensor package, bonding condition, and changing boundary state, rather than as a calibrated shaft speed or absolute acceleration signal. Adaptive variational mode decomposition (AVMD) was applied to track the time-varying narrowband spectral-response trajectory of the Bragg-wavelength signal. In parallel, raw wavelength windows were supplied to LSTM, 1D-CNN, and CNN–LSTM autoencoders to evaluate waveform departures from learned nominal fixed-angle behavior. The fixed-angle results showed stable but distinguishable optical vibration responses under different boundary states, whereas the dynamic angle-transition records produced local trajectory changes and alarm-candidate intervals. Baseline and autoencoder comparisons further clarified the trade-off between transition coverage and false-alarm tendency. The RMS threshold baseline was more sensitive to transition-related amplitude changes but produced more false alarms, whereas the CNN–LSTM autoencoder provided the most selective response among the tested autoencoder branches. The results are interpreted as task-specific evidence for relative vibration-state transition monitoring rather than as general motor fault diagnosis. Overall, the framework demonstrates a compact FBG-based route for relative vibration-state transition monitoring when speed references, dense sensor layouts, and labeled fault data are unavailable.

## 1. Introduction

Vibration measurements are widely used to observe the operating behavior of rotating and motor-driven machinery. Changes in the time waveform, spectral content, and transient vibration response can provide useful information for machinery monitoring and diagnosis [1,2,3,4]. In many practical installations, however, the sensor is not placed directly on the rotating shaft or bearing seat. It is often attached to a support frame, housing, fixture, or external structure where the measured signal is shaped by the vibration source, mechanical transfer path, mounting condition, and environmental boundary. Under changing inclination or support conditions, the recorded waveform may therefore reflect a boundary-dependent vibration state rather than a direct measurement of a single internal mechanical component. This situation is particularly relevant for compact motor–support assemblies, mobile platforms, inclined equipment, and remote monitoring scenarios in which tachometers, dense sensor layouts, or labeled fault data are not available [5,6,7]. Practical examples include end-effector motors on robotic arms, propulsion or steering modules in mobile robots, inclined conveyor systems, and compact motors mounted on adjustable supports. In these cases, the measured vibration response may change even under nominally unchanged motor commands because the support path, gravity direction, and fixture coupling are altered.

Fiber Bragg grating (FBG) sensors are attractive for such monitoring tasks because they are compact, passive, wavelength-encoded, and suitable for remote optical interrogation [8,9,10]. These properties are useful near motors and electrical equipment, where electromagnetic interference, wiring complexity, and access limitations can restrict the use of conventional electrical sensors. Nevertheless, when a packaged FBG sensor is attached to a motor–support structure, the resulting Bragg-wavelength fluctuation should be interpreted carefully. The measured optical signal contains vibration-induced strain transmitted through the motor, fixture, sensor package, bonding layer, and mounting boundary. Without an independent speed reference or acceleration calibration, the Bragg-wavelength fluctuation is interpreted in this study as a relative vibration-sensitive optical response of the coupled motor–fixture–sensor package–boundary system.

**Figure 1:**
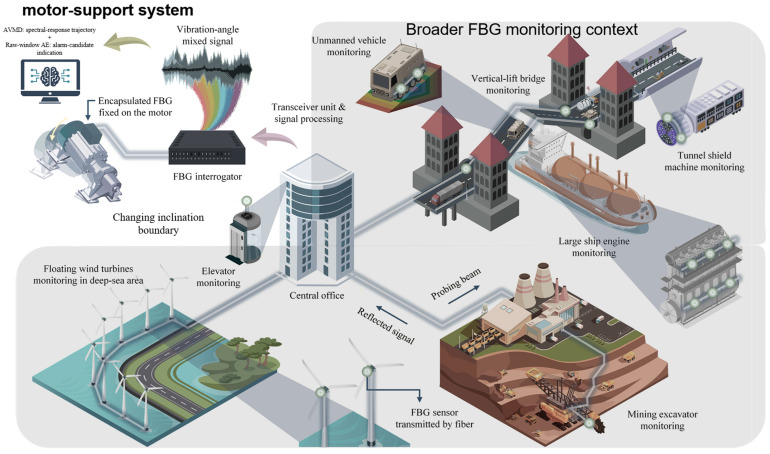
Study-specific grating-based fiber-optic sensing path for boundary-dependent motor vibration-state transitions, linking packaged FBG interrogation with AVMD spectral tracking and raw-window autoencoder alarm indication.

Figure 1 illustrates the study-specific FBG remote sensing architecture and analysis path. Although FBG sensors can be deployed in different industrial and structural monitoring scenarios, this work is restricted to a single packaged FBG sensor mounted on a motor–support structure under angle-dependent boundary conditions. The demodulated Bragg-wavelength sequence is treated as a coupled vibration-sensitive optical response rather than as a calibrated shaft speed or absolute acceleration signal. This single-channel signal is interpreted through an AVMD-based spectral-response branch and a raw-window autoencoder alarm branch for relative vibration-state transition monitoring.

This study asks whether a single packaged FBG channel can provide relative vibration-state information for a motor–support system under angle-dependent boundary conditions. The objective is not a categorical motor fault diagnosis, but observation of fixed-boundary vibration-state patterns and dynamic boundary-transition responses from a single optical channel. The proposed framework combines an AVMD-based spectral interpretation branch with a raw-window autoencoder alarm branch to track narrowband spectral-response trajectories and identify waveform departures from learned nominal fixed-angle behavior.

FBG-based vibration and acceleration sensing have been widely investigated, including applications in vibration sensing, acceleration measurement, low-frequency vibration detection, high-precision vibration sensing, and electric machinery monitoring [11,12,13,14,15,16,17,18]. Recent studies have also explored AI-assisted FBG sensing and single-FBG temperature–vibration decoupling, showing the potential of combining optical sensing with data-driven interpretation [14,19]. However, the interpretation of a single-channel FBG signal becomes more subtle when the sensor is mounted on the motor support or housing rather than directly on the rotating shaft. In that configuration, the Bragg-wavelength fluctuation is a vibration-sensitive optical response of the coupled system. It may contain information related to motor operations, support-path variations, and boundary-dependent vibration transfer. Therefore, in the absence of an independent tachometer or acceleration calibration, this study interprets the wavelength-domain response as a relative optical indicator of motor–support vibration behavior. This caution is consistent with tacholess rotating-machinery analysis, where frequency-like trajectories must be interpreted carefully when no tachometer or speed reference is available [20].

Our previous work on FBG-based robotic sensing showed that dynamic strain signals can support learning-based activity recognition in mechanical robotic platforms [21]. The present study shifts the emphasis from activity labeling to relative vibration-state monitoring of a motor under angle-dependent boundary conditions. Instead of constructing a feature-heavy pipeline based on hand-selected spectral peaks or mode-energy ratios, this work combines an interpretation-oriented AVMD branch with a raw-window autoencoder alarm branch. The intention is to keep the physical observation visible while allowing the monitoring output to remain data driven.

For the interpretation branch, AVMD is used to follow the time-varying narrowband spectral-response trajectory of the Bragg-wavelength signal. Compared with fixed-resolution time–frequency methods, the AVMD branch was selected because it can adaptively separate nonstationary Bragg-wavelength responses into narrowband components and provide a compact trajectory for tracking boundary-dependent spectral movement. Variational-mode-based decomposition and adaptive variants provide a useful basis for separating nonstationary signals into band-limited components and tracking modal behavior over time [22,23,24,25]. In this study, the AVMD-tracked trajectory is not presented as a direct motor-speed measurement. It is used as a relative spectral descriptor that helps reveal how the vibration-sensitive optical response evolves when the mechanical boundary changes. This role is important because the raw wavelength waveform can look dense and visually similar across operating intervals, whereas a narrowband trajectory can make boundary-dependent changes more interpretable.

For the alarm branch, raw Bragg-wavelength windows are supplied directly to autoencoder models. Autoencoders have been used for anomaly and deviation detection because they can learn a nominal representation from normal data and highlight inputs that are reconstructed less consistently with that learned manifold [26,27,28,29,30,31,32]. Here, LSTM and CNN–LSTM autoencoder variants [27,33,34], together with a 1D-CNN AE branch, are used to examine whether local waveform structure and short-range temporal continuity can support transition-sensitive monitoring. Reconstruction behavior and latent-transition changes are converted into alarm-candidate indications when the waveform departs from the learned nominal vibration-state manifold. The resulting alarm output is therefore positioned as a compact indication of relative vibration-state change, aligned with the available single-channel optical measurement.

By combining these two branches, this study presents a grating-based fiber-optic sensing framework for relative motor vibration-state transition monitoring under angle-dependent boundary conditions. The AVMD branch provides an interpretable view of the evolving narrowband spectral response, while the raw-window autoencoder branch provides an alarm-candidate output for waveform departures from learned nominal behavior. The experimental demonstration focuses on representative fixed-boundary and dynamic boundary-change observations under the same nominal motor drive setting. This work demonstrates how a packaged FBG sensor, an AVMD-based spectral interpretation path, and a raw-window alarm-candidate learning path can be integrated into a compact remote monitoring scheme for situations in which conventional speed references, dense sensor layouts, or labeled fault data are difficult to obtain.

The main contributions of this study are summarized as follows. First, a single packaged FBG sensing channel is used to observe relative motor–support vibration states under controlled angle-dependent boundary conditions. Second, AVMD is used as an interpretation branch to reveal boundary-dependent changes in the narrowband spectral-response trajectory that are not easily distinguished from the dense raw Bragg-wavelength waveform alone. Third, raw-window autoencoder models, including LSTM AE, 1D-CNN AE, and CNN–LSTM AE, are compared as alarm-branch candidates for detecting waveform departures from learned nominal fixed-angle behavior. Fourth, RMS threshold and PCA-reconstruction-error baselines are included to clarify the trade-off between sensitivity, false-alarm tendency, and alarm selectivity. The resulting framework is positioned as a single-channel relative vibration-state transition monitoring approach, with emphasis on boundary-dependent sensing behavior and transition-sensitive alarm indication.

## 2. Work Principle and Experimental Setup

The experimental design was guided by a sensing-oriented question: whether a single packaged FBG sensing channel, installed away from the rotating shaft, can provide relative information about the vibration state of a motor–support system when the mechanical boundary condition changes. In many practical installations, the accessible sensing position is not the shaft, bearing seat, or internal winding, but a support frame, housing, or fixture. The optical waveform recorded at this location is therefore a coupled response of the motor, sensor package, mounting structure, fixture coupling, and inclination-dependent load path. This mixed response is treated as the object of monitoring in the present study. The experiment evaluates whether the single FBG channel can reveal fixed-boundary vibration-state patterns and dynamic boundary-transition responses from the coupled motor–support system.

The sensing element was a packaged single-axis FBG accelerometer-type unit mounted on the support structure of a compact AC motor using a fixture-based coupling arrangement. The motor was an AC-powered compact motor in the approximate 850 W class. The packaged FBG sensing unit had a nominal acceleration range of approximately ±10 m/s^2^. During operation, the reflected Bragg wavelength was demodulated by an FBG interrogation unit at a sampling rate of 200 Hz. The interrogator had a wavelength accuracy of 10 pm and a dynamic range of 25 dB. The resulting data stream was a one-dimensional Bragg-wavelength sequence, where the wavelength fluctuations reflected vibration-induced strain variations transmitted through the motor–support assembly. Because an independent wavelength-to-acceleration calibration was not performed in this study, the wavelength-domain response was analyzed directly rather than converted into absolute acceleration.

The sensing principle relies on the wavelength-encoded response of the FBG. When vibration is transferred to the packaged sensing structure, the strain acting on the grating modifies its optical response and produces a measurable Bragg-wavelength fluctuation. Because the sensing head is passive and connected through an optical fiber, the measurement point can be separated from the interrogation and computing unit. This configuration is useful for motor-related monitoring because the sensor can remain compact and electrically passive near the machine, while the demodulation and analysis hardware can be placed at a safer location. In this experiment, all interpretations were based on the raw Bragg-wavelength waveform. No tachometer, electrical current reference, conventional accelerometer, or multi-axis sensor array was used.

The motor was installed on an adjustable-angle platform to change the mechanical boundary condition relative to gravity. Throughout the experiments, the nominal motor drive setting and sensor mounting configuration were kept unchanged so that the optical response could be compared under different boundary configurations without intentionally changing the drive command or sensor coupling. The measured wavelength fluctuation is therefore interpreted as a vibration-sensitive optical response of the coupled system. This formulation is important because the same nominal drive condition may produce different observed responses when the support path, inclination, or load direction changes.

The packaged FBG sensing unit was used to observe vibration-sensitive responses transmitted through the motor–support structure. Therefore, the experiment is positioned as a low-frequency relative vibration-state monitoring task rather than a complete motor fault diagnosis study. The analysis is suitable for observing structural motion, support-path changes, vibration-state variations, and boundary-transition effects. However, without additional high-frequency vibration instrumentation or independent acceleration calibration, the present framework is not intended to evaluate high-frequency diagnostic components that may be important in conventional motor fault diagnosis, such as bearing-defect-related components, high-frequency resonance responses, or electrical harmonics. For this reason, the present results are interpreted within the scope of relative vibration-state transition monitoring under controlled boundary changes.

Two experimental protocols were used in the main analysis. The first consisted of fixed-angle measurements at representative inclination conditions. The selected fixed angles were chosen to provide stable boundary states within the tested operating range of the adjustable motor–support platform. These measurements provided steady records under controlled boundary conditions. Their purpose was not to construct a complete angle–frequency calibration map, but to examine whether the single FBG channel could capture distinguishable relative vibration responses when the support geometry was changed. Each fixed-angle record was acquired under the same nominal motor drive setting, allowing the comparison to focus on the influence of the boundary condition and sensing transfer path.

The second protocol introduced dynamic angle-transition conditions. In these tests, the motor–support platform was gradually moved between low- and high-inclination states while the nominal drive condition remained fixed. These records created nonstationary vibration responses in which the boundary condition evolved during operation. Compared with the fixed-angle records, the transition sequences were expected to contain short-lived waveform changes, local spectral movement, and changes in the vibration transfer path. They therefore provided suitable cases for evaluating whether the analysis framework could highlight relative state changes beyond steady-state differences.

All experiments were conducted under a fixed-temperature laboratory environment. The raw Bragg-wavelength sequences were stored without conversion into handcrafted physical descriptors in this step. The fixed-angle records served as nominal operating data for learning representative steady vibration behavior, whereas the dynamic transition records were used to observe time-varying response changes. The present analysis focused on short-term vibration-state responses associated with fixed-angle and dynamic boundary-transition conditions. Because FBGs are inherently sensitive to both strain and temperature, the temperature effect was considered when interpreting the measured Bragg-wavelength response. No separate temperature-compensation grating or temperature-decoupling algorithm was implemented in this study. Instead, the influence of temperature was minimized under the fixed-temperature laboratory condition, and the analysis focused on short-time vibration-related wavelength fluctuations rather than long-term thermally induced Bragg-wavelength drift. Therefore, the reported results are interpreted as relative vibration-state indicators rather than temperature-compensated acceleration measurements. Future work will include a strain-free reference FBG or an additional temperature channel for long-term field deployment.

In the following methodology, the same raw optical signal is examined through two complementary branches: an AVMD branch for interpreting the narrowband spectral-response trajectory and a raw-window autoencoder branch for alarm-candidate indication based on waveform departure from learned nominal behavior.

## 3. Methodology

### 3.1. Data Collection and Data Preprocessing

The recorded optical data consisted of a one-dimensional Bragg-wavelength time series continuously demodulated by the FBG interrogator at a sampling rate of 200 Hz. Each record, therefore, represented the temporal vibration-sensitive response of the motor–fixture–boundary system as observed through a single optical sensing channel. Before being supplied to the analysis models, the continuous wavelength sequence was segmented into overlapping raw-data windows. Each window contained 100 consecutive samples, corresponding to a duration of 0.5 s, and adjacent windows were extracted with an 80% overlap. The resulting step size was 20 samples, which provided a dense temporal description of the waveform evolution while preserving short-term continuity between neighboring windows.

Using this segmentation strategy, 10,704 windows were obtained for model training, and 10,033 windows were reserved for testing. These numbers refer to overlapping signal windows rather than independent experimental trials. In other words, multiple windows were extracted from each continuous measurement record, and the window count should be interpreted as the number of analysis units used by the signal-processing and learning pipeline. This clarification is important because the purpose of the windowing procedure was not to artificially increase the number of independent experiments, but to transform a continuous Bragg-wavelength record into a sequence of locally comparable waveform segments.

The fixed-angle records were used to represent nominal steady vibration states under controlled inclination-dependent boundary conditions. These data formed the training set for the autoencoder models. The dynamic angle-transition records were held out for testing, allowing the trained models to evaluate waveform departures under nonstationary boundary-change conditions. During training, no fault labels, speed references, external accelerometer signals, or manually assigned transition labels were provided to the models. Each input was treated simply as a raw Bragg-wavelength window associated with the measured optical vibration response.

No handcrafted descriptors, such as the root mean square value, standard deviation, selected spectral peak, or mode-energy ratio, were computed as model inputs. Instead, each 100-sample Bragg-wavelength window was supplied directly to the autoencoder branch. This design keeps the learning process close to the measured optical waveform and avoids imposing a predefined physical feature set on a signal that is inherently affected by motor vibration, fixture dynamics, bonding conditions, and angle-dependent load transfer. The high overlap between neighboring windows also supports the latent-transition analysis. Under nominal steady conditions, consecutive windows share most of their samples and are expected to produce similar latent representations. When the waveform changes more abruptly during a boundary transition, the corresponding latent representation is more likely to depart from the previous state, making the latent-transition score a compact indicator of relative vibration-state change.

### 3.2. AVMD-Assisted Spectral-Response Interpretation

AVMD was used as an interpretation-oriented branch to examine how the vibration-sensitive Bragg-wavelength response evolved under different motor boundary conditions. This branch provides a consistent spectral-response view of the nonstationary optical waveform while keeping the physical observation visible. Because the FBG sensor was mounted on the motor–support structure, the measured signal contained the coupled system response rather than a direct shaft or bearing response. The AVMD-tracked trajectory was therefore interpreted as a relative spectral descriptor of this coupled optical signal.

To ensure consistent processing across records, the same AVMD configuration was used for all displayed records. The Bragg-wavelength signal was sampled at 200 Hz. The candidate VMD modes were searched adaptively with an upper limit of K_max = 25, while the bandwidth penalty parameter was kept fixed throughout the analysis. In this study, adaptive refers to the mode-number search rather than to a record-specific adjustment of the bandwidth penalty. The AVMD-tracked trajectory was interpreted only within the usable vibration-response range of the packaged FBG sensing unit and the controlled experimental configuration. Therefore, the AVMD output was used as a relative narrowband spectral-response descriptor rather than as a complete broadband vibration reconstruction.

The mode used for trajectory tracking was selected according to spectral concentration, temporal continuity of the extracted ridge, and consistency with the dominant vibration-sensitive response observed in the Bragg-wavelength record. Modes mainly representing near-DC drift, broadband residuals, or isolated components outside the usable response range of the sensing configuration were excluded from the displayed trajectory. Amplitude- and spectral-validity criteria were applied to suppress low-energy intervals and isolated short-lived spectral peaks. Because a large bandwidth penalty may suppress broadband or short-lived components, the AVMD trajectory is used only as a relative narrowband spectral descriptor rather than as a complete broadband vibration reconstruction.

The final AVMD output was the time-varying spectral-response trajectory of the selected vibration-sensitive mode. In the fixed-angle records, this trajectory was used to compare whether the optical vibration response remained stable and distinguishable under controlled inclination-dependent boundary states. In the dynamic angle-transition records, the same procedure was used to observe whether the narrowband response exhibited temporary movement during boundary changes. These trajectory variations were interpreted as changes in the vibration-transfer path and relative vibration state of the coupled system.

### 3.3. Raw-Window Autoencoder and Latent-Transition Alarm Logic

The alarm branch was designed to evaluate whether a raw Bragg-wavelength window remained consistent with the learned nominal vibration-state behavior. A continuous Bragg-wavelength sequence was first divided into overlapping raw-data windows and supplied to three compact autoencoder structures: an LSTM AE, a 1D-CNN AE, and a hybrid CNN–LSTM AE. These three models were included to clarify the role of different waveform-learning mechanisms. The LSTM AE modeled short-range temporal continuity, the 1D-CNN AE extracted local waveform patterns, and the CNN–LSTM AE combined local fluctuation extraction with sequential encoding.

**Figure 2:**
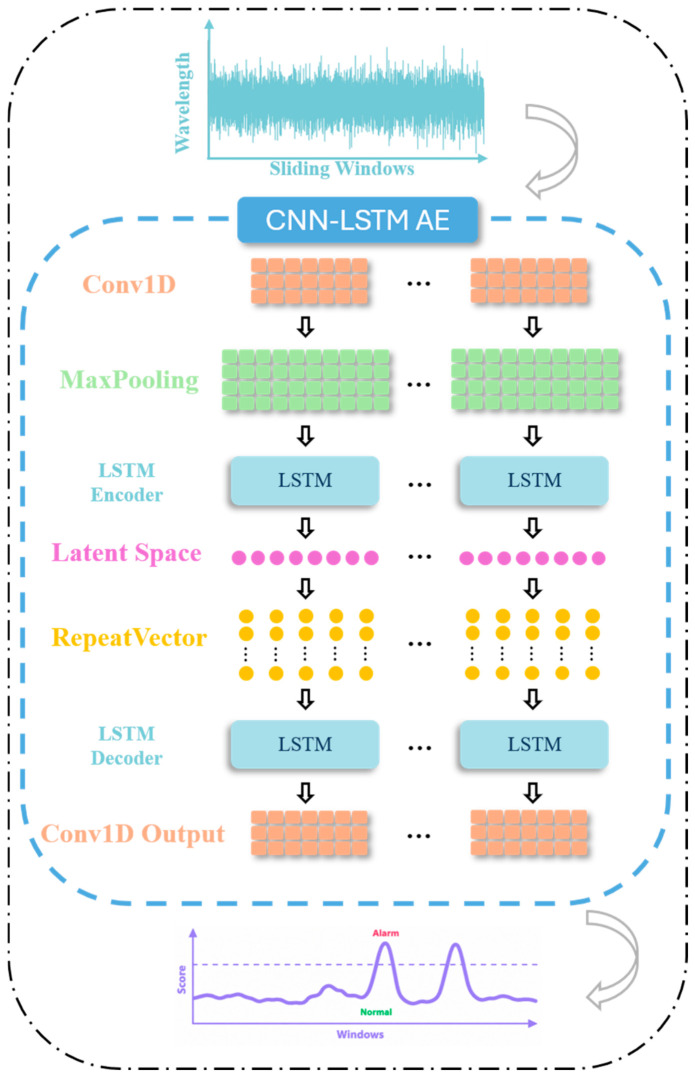
Representative CNN–LSTM autoencoder structure for raw-window Bragg-wavelength reconstruction and latent-transition alarm-candidate indication.

Figure 2 shows the representative CNN–LSTM autoencoder structure used in the raw-window alarm branch. The input Bragg-wavelength sequence is first segmented into sliding windows. In the encoder, convolutional and pooling layers extract compact local fluctuation patterns from the raw wavelength window, and the LSTM layer maps the encoded sequence into a latent representation. The decoder then reconstructs the input window through a repeat-vector operation, LSTM decoding, and one-dimensional convolutional output. The reconstruction behavior and latent-space movement are subsequently used to generate alarm-candidate indications. All models reconstructed the same 100-sample Bragg-wavelength window and were trained using reconstruction loss with the Adam optimizer; nominal validation data were used to set the alarm thresholds.

Training was performed without fault labels, transition labels, external speed references, or additional sensor channels. No handcrafted descriptors, such as RMS, standard deviation, selected spectral peak, or mode-energy ratio, were used as model inputs. After training, each test window was reconstructed by the corresponding autoencoder. The reconstruction error was used as a compact alarm index: a larger error indicates that the incoming waveform is less consistent with the learned nominal vibration-state manifold. In addition, encoded latent vectors were used to calculate a latent-transition score between neighboring windows. Because adjacent windows share a large portion of their samples, nominal steady operation is expected to produce similar latent representations, whereas boundary-transition-induced waveform changes may produce localized score increases.

A high-quantile threshold, defined here as the 99th percentile of the nominal validation score distribution, was applied to the reconstruction and latent-transition responses. Windows exceeding the corresponding threshold were marked as alarm candidates and interpreted as relative vibration-state departures from learned nominal behavior. A meaningful transition may therefore appear as a short interval of neighboring alarm candidates rather than as a single isolated point.

For evaluation only, transition labels were assigned post hoc from the held-out dynamic transition records using a 50-sample rolling-standard-deviation change threshold at the 99th percentile, followed by three-sample mask dilation and window-level conversion. These labels were not used during autoencoder training and were used only to compute precision, recall, F1-score, false-alarm rate, and latency. The false-alarm rate was calculated as the fraction of non-transition windows classified as alarm candidates. Latency was defined as the time difference between the onset of the post hoc transition interval and the first subsequent alarm-candidate window.

For baseline comparison, two non-neural alarm methods were implemented using the same raw-window segmentation and nominal validation thresholding strategy. The first baseline was an RMS threshold alarm. For each 100-sample Bragg-wavelength window, the window mean was removed, and the root mean square value of the residual wavelength fluctuation was used as an amplitude-based alarm score. The second baseline was a PCA reconstruction-error alarm. The nominal fixed-angle windows were used to estimate a PCA subspace, and each validation or test window was projected into and reconstructed from this subspace. The PCA subspace was determined using a 95% cumulative explained-variance criterion based on the nominal training windows. The reconstruction error was then used as the PCA alarm score. Both baseline thresholds were derived from the corresponding nominal validation score distributions, and the resulting alarm outputs were evaluated using the same precision, recall, F1-score, false-alarm rate, and latency definitions as the autoencoder models.

## 4. Results and Discussion

### 4.1. Fixed-Angle Vibration Response Under Same Nominal Motor Setting

**Figure 3:**
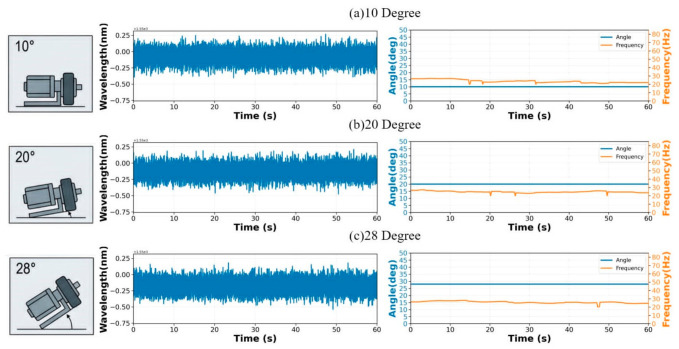
Representative fixed-angle Bragg-wavelength responses and AVMD-tracked spectral-response trajectories at 10°, 20°, and 28° under identical nominal motor drive settings.

Under the same nominal motor drive setting, the three fixed-angle records provide a controlled view of how the FBG sensing path responds when only the inclination-related boundary condition is changed. The raw Bragg-wavelength waveforms in Figure 3 show continuous vibration-induced fluctuations for all three angles. No obvious clipping, saturation, or isolated burst-like disturbance is observed within the displayed records, suggesting that the interrogated wavelength response remained stable during the fixed-angle measurements. The waveform amplitudes are not identical among the three cases, indicating that the measured coupled response varies with the inclination-related boundary condition.

A useful observation from the raw traces is that the time-domain Bragg-wavelength signal alone provides only a dense and visually similar vibration pattern. Although the fixed-angle records are steady in the sense that the motor drive condition is unchanged, the measured optical waveform still carries the influence of the support geometry and load-transfer path. This is consistent with the experimental configuration, where the FBG sensor was attached to the motor–support structure rather than to the rotating shaft. The recorded wavelength fluctuation, therefore, represents the vibration-sensitive optical response at the selected support-structure sensing location.

The AVMD-tracked spectral-response trajectories provide a compact way to compare these steady boundary states. For each fixed-angle case, the tracked trajectory remains relatively stable over time, indicating that the selected narrowband response does not undergo strong nonstationary movement during the fixed-angle interval. At the same time, the trajectory levels are not exactly identical across the three inclination conditions. This observation suggests that the single FBG channel captures angle-dependent changes in the relative vibration state of the motor–support system. The trajectory is therefore read as a relative spectral descriptor of the measured optical response under controlled boundary conditions.

This distinction is important for the interpretation of Figure 3. The purpose of the fixed-angle comparison is to establish representative steady reference patterns under controlled boundary conditions, rather than to construct a complete angle–frequency calibration curve. These patterns define the nominal vibration-state behavior used later by the raw-window autoencoder branch. In this sense, the fixed-angle results form the baseline layer of the proposed framework: the raw wavelength traces confirm that the FBG sensor captures stable vibration-related optical fluctuations, while the AVMD trajectories provide a concise representation of how the relative vibration state differs among boundary configurations.

The wavelength fluctuation range observed in the raw traces can be discussed qualitatively as remaining within a stable measurement regime. Quantitative conversion from Bragg-wavelength variations to acceleration amplitude would require a calibrated wavelength-to-acceleration sensitivity for the packaged FBG accelerometer. Therefore, the present analysis emphasizes relative vibration-state observation and the consistency of the sensing response under repeated steady motor operation.

### 4.2. Dynamic Angle-Transition Tracking Under Fixed Motor Drive Setting

**Figure 4:**
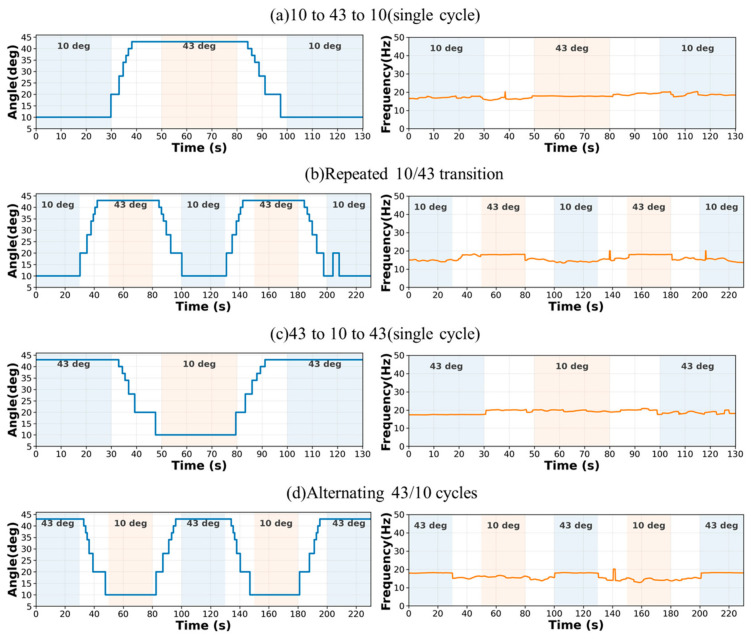
Dynamic angle-transition profiles and AVMD-tracked spectral-response trajectories under identical nominal motor drive settings.

The dynamic angle-transition records provide a more demanding test than the fixed-angle measurements because the mechanical boundary condition changes during operation while the nominal motor drive setting remains unchanged. In this condition, the measured optical vibration response is expected to contain both steady vibration components and transient boundary-induced variations. The angle profiles and the corresponding AVMD-tracked spectral-response trajectories in Figure 4 show how the narrowband optical response evolves when the motor–support platform moves between low- and high-inclination states.

A clear observation is that the tracked spectral-response trajectory does not behave as a simple angle readout. During intervals in which the angle remains approximately constant, the AVMD trajectory tends to stay within a relatively narrow band, suggesting that the selected narrowband response is stable under a fixed-boundary state. When the angle changes, however, the trajectory may exhibit short-lived movement, local fluctuations, or spike-like disturbances. These local changes are especially meaningful because the motor drive condition is not intentionally changed. The observed response is therefore associated with the changing vibration-transfer path of the coupled system.

The dynamic transition results further clarify why a time-varying spectral descriptor is useful for this experiment. A single fixed-angle record can describe a steady relative vibration state, whereas the dynamic transition records reveal how the response moves between boundary states. The AVMD-tracked trajectory provides a compact view of how the optical vibration response reorganizes when the support boundary changes. This interpretation is consistent with the single-channel sensing configuration, where the FBG sensor is mounted on the support structure instead of the rotating shaft.

The transient movements in the trajectory are read as transition-sensitive spectral responses associated with boundary-induced changes in the vibration-transfer path. In practical monitoring, such behavior is valuable because it marks the time interval in which the vibration state departs from the steady fixed-angle pattern. This provides the physical motivation for the subsequent autoencoder analysis: if the AVMD branch makes the boundary-induced spectral movement visible, the raw-window autoencoder branch can convert related waveform departures into alarm-candidate intervals. The dynamic transition results show that the proposed framework is able to follow relative vibration-state changes under changing boundary conditions using only the measured single-channel optical response.

### 4.3. AI-Based Latent-Transition Alarm Demonstration

**Figure 5:**
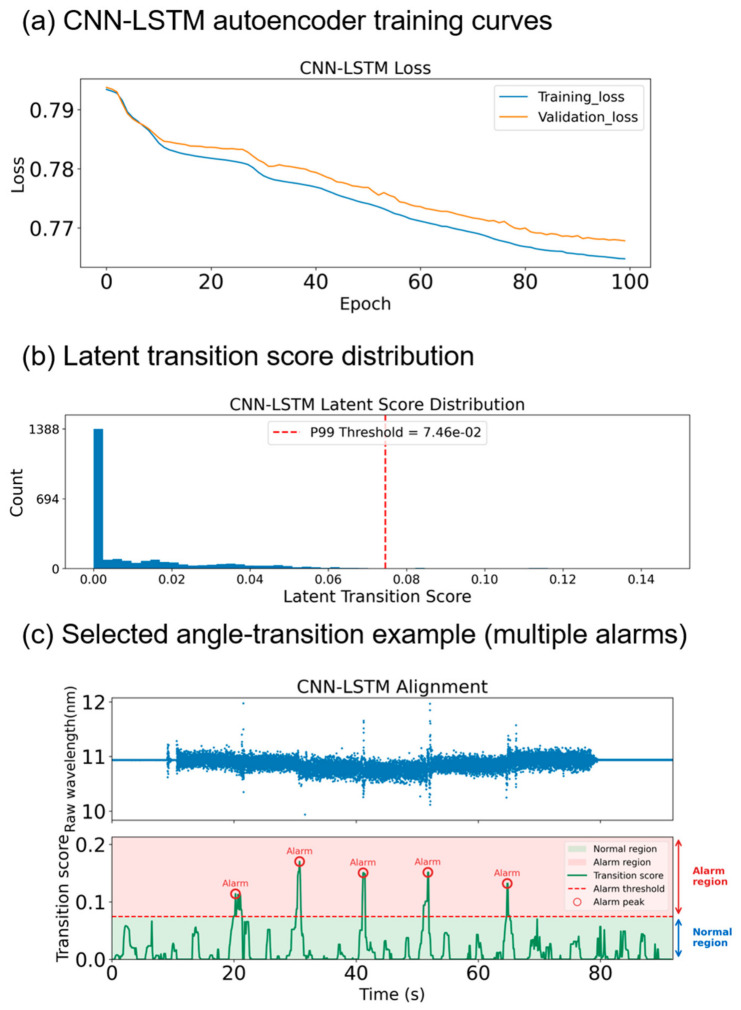
Autoencoder training behavior and latent-transition alarm demonstration for the representative angle-transition case. (**a**) Training and validation loss curves of the CNN–LSTM autoencoder. (**b**) Latent-transition score distribution with a 99th-percentile threshold. (**c**) Raw Bragg-wavelength sequence and latent-transition alarm-candidate response.

The autoencoder branch was used to convert waveform-level departures into a compact monitoring output. After the CNN–LSTM autoencoder was trained on nominal fixed-angle windows, its training behavior was first examined through the training and validation loss curves. As shown in Figure 5a, both losses decrease gradually and approach a stable range over the training epochs. The validation curve remains close to the training curve, suggesting that the model learned the main structure of the nominal Bragg-wavelength windows without producing an obvious divergence between training and validation behavior. This observation supports the use of the learned representation as a nominal vibration-state reference for subsequent transition monitoring.

The latent-transition score distribution provides the next layer of interpretation. Most scores are strongly concentrated near zero, which is an expected outcome of the sliding-window design. Since adjacent windows have high overlap, neighboring nominal windows share most of their waveform content and tend to produce similar latent representations. The long-tailed portion of the distribution is therefore more informative than the main cluster: it defines the region where the latent representation changes more strongly than an ordinary nominal-window variation. A high-quantile threshold was applied to this distribution to form a conservative boundary for alarm-candidate selection, as illustrated in Figure 5b.

The selected angle-transition example further shows how this score behaves when the boundary condition changes. In Figure 5c, the raw Bragg-wavelength sequence remains densely oscillatory, and the transition-related behavior is not immediately separable from the waveform alone. The latent-transition score, however, produces several localized peaks that exceed the threshold. These peaks are better read as short transition-sensitive intervals around the boundary change, where the raw waveform departs from the learned nominal vibration-state manifold. Because the input windows overlap, neighboring alarm candidates may occur around the same physical transition and are better interpreted as a transition-sensitive alarm interval.

This result complemented the AVMD interpretation branch. AVMD makes the time-varying narrowband spectral response visible, while the autoencoder branch provides a threshold-based indication of when the raw waveform becomes less consistent with nominal fixed-angle behavior. The agreement between localized latent-score peaks and the angle-transition interval suggests that the proposed raw-window approach can support relative vibration-state monitoring directly from the measured single-channel optical waveform. The alarm output is therefore positioned as an early indication of waveform departure under changing boundary conditions.

### 4.4. Autoencoder Model Comparison for Transition-Sensitive Alarm Indication

**Figure 6:**
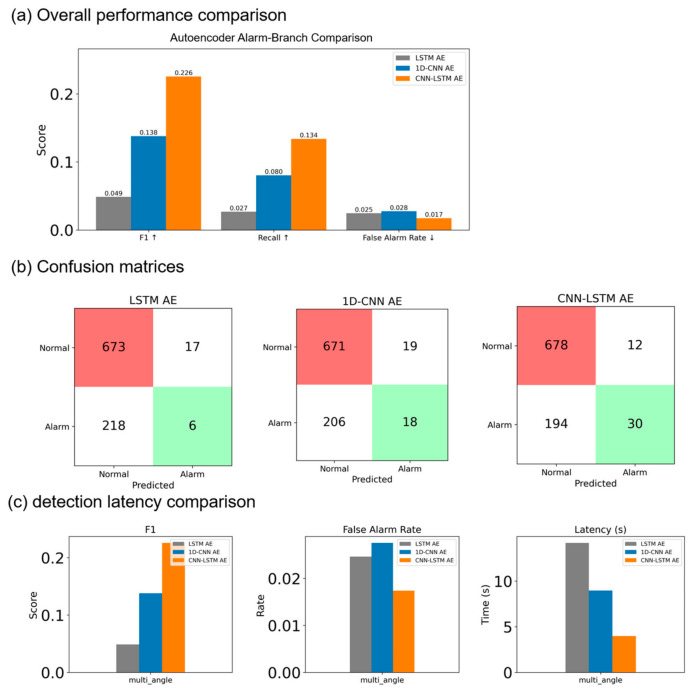
Autoencoder alarm-branch comparison for transition-sensitive indication. (**a**) Summary of window-level autoencoder alarm performance using F1-score, recall, and false-alarm rate. (**b**) Window-level alarm confusion matrices for LSTM AE, 1D-CNN AE, and CNN–LSTM AE. The horizontal axis denotes the predicted label, and the vertical axis denotes the true label. (**c**) Detection latency comparison. Detailed numerical values for the three autoencoder candidates are listed in Table 1.

The comparison in this section first focuses on the three autoencoder alarm-branch candidates shown in Figure 6 and Table 1: LSTM AE, 1D-CNN AE, and CNN–LSTM AE. Two non-neural baseline methods, an RMS threshold alarm and a PCA reconstruction-error alarm, are then discussed in the main text to provide additional context for interpreting the autoencoder results. Because Figure 6 presents the autoencoder alarm-branch comparison, the RMS and PCA baselines are treated as reference methods for sensitivity-oriented and reconstruction-based alarm behavior.

For the autoencoder comparison, the objective was to identify windows or intervals whose Bragg-wavelength waveform departed from the learned nominal fixed-angle vibration-state behavior. Accordingly, the comparison focused on precision, recall, F1-score, false-alarm rate, and alarm latency. As summarized in Figure 6a and Table 1, the three autoencoder alarm-branch candidates showed different trade-offs in transition coverage, false-alarm tendency, and latency. The LSTM AE showed the weakest transition coverage, with an F1-score of 0.049 and a recall of 0.027. This result suggests that temporal modeling alone was not sufficient to capture the transition-sensitive waveform departures in the single-channel FBG record. The 1D-CNN AE improved the F1-score to 0.138 and the recall to 0.080, indicating that local Bragg-wavelength fluctuation patterns are useful for detecting waveform departures during boundary transition. However, the 1D-CNN AE also produced the highest false-alarm rate among the three tested autoencoder models in this dynamic evaluation.

Among the three autoencoder branches, the CNN–LSTM AE provided the most selective transition-sensitive response. It achieved the highest precision of 0.714, an F1-score of 0.226, and a recall of 0.134 among the autoencoder models, while also producing the lowest false-alarm rate of 0.017 and the shortest autoencoder latency of 3.99 s. This trend suggests that combining local waveform extraction with short-range temporal modeling is useful for the present boundary-transition FBG monitoring task. The convolutional component captures local fluctuation patterns within the raw Bragg-wavelength window, whereas the LSTM component preserves the temporal organization of the encoded features.

Because Table 1 lists the detailed numerical values of the three autoencoder candidates shown in Figure 6, the RMS threshold and PCA reconstruction-error baselines are reported quantitatively in the following text. The RMS threshold baseline used the root mean square value of the mean-removed Bragg-wavelength fluctuation in each raw window as an amplitude-based alarm score. It achieved a precision of 0.448, a recall of 0.500, an F1-score of 0.473, a false-alarm rate of 0.200, and a latency of 1.77 s. The higher F1-score and recall of the RMS threshold baseline indicate that the dominant transition response in the present dynamic evaluation contained an amplitude-sensitive component. However, its higher false-alarm rate indicates greater susceptibility to nominal background amplitude variations.

The PCA reconstruction-error baseline used the reconstruction error after projecting each window into the nominal PCA subspace and reconstructing it back to the original window space. The PCA subspace was determined using a 95% cumulative explained-variance criterion based on the nominal training windows. This baseline achieved a precision of 0.059, a recall of 0.009, an F1-score of 0.016, a false-alarm rate of 0.046, and a latency of 16.05 s, suggesting that a linear reconstruction subspace did not effectively capture the transition-sensitive waveform departures in the present single-channel FBG record.

The relatively low recall and F1-score of the autoencoder models should be interpreted in relation to the window-level evaluation setting and the conservative 99th-percentile threshold. In the present dynamic transition records, boundary-change intervals are short compared with the surrounding nominal windows. Therefore, a selective alarm branch may miss some transition-labeled windows while still producing localized alarm candidates within the transition interval. From a monitoring perspective, such clustered alarm candidates are useful for highlighting transition-sensitive intervals, although the window-level recall remains limited. The RMS threshold baseline is therefore useful as a sensitivity-oriented reference method, whereas the CNN–LSTM AE should be interpreted as a complementary selectivity-oriented alarm branch rather than a generally superior replacement for RMS-based monitoring.

For practical intelligent sensing applications, robustness is important because monitoring signals may be affected by sensor noise, mounting variations, operating-condition changes, environmental drift, and sensor faults. Recent studies on physical system input–output monitoring and industrial time-series anomaly detection have emphasized the effects of multiple interfering factors, limited labels, changing conditions, and distribution shifts [35,36,37]. These issues are relevant here because the measured Bragg-wavelength response reflects not only motor vibration but also fixture dynamics, sensor-package coupling, bonding condition, and boundary-dependent load transfer.

In this study, robustness was considered through a limited comparison of alarm sensitivity and selectivity using the single-channel FBG boundary-transition records. The comparison shows that different alarm strategies emphasize different monitoring priorities. The RMS threshold alarm is useful when transition sensitivity and fast response are prioritized, but it may require additional filtering or persistence logic to reduce false alarms. The PCA reconstruction-error baseline was less effective for this dynamic transition evaluation, suggesting that a linear reconstruction model was insufficient for capturing the relevant waveform departures. The CNN–LSTM AE may be more suitable when a lower false-alarm tendency and higher alarm selectivity are prioritized. Therefore, the reported alarm behavior should be interpreted as task-specific evidence for this controlled boundary-transition evaluation rather than as proof of general industrial robustness.

This evaluation does not represent a complete industrial robustness validation. Further tests with repeated trials, different loading conditions, sensor remounting, temperature variations, long-term drift, changing motor speeds, and sensor-fault scenarios are still needed before practical deployment.

Figure 6b presents representative window-level alarm confusion matrices for the three autoencoder candidates. These matrices visualize how nominal and alarm-candidate windows are distributed after thresholding. The CNN–LSTM AE shows fewer nominal windows classified as alarm candidates and more transition-related alarm candidates than the 1D-CNN AE in the displayed representative case. This observation is consistent with the lower false-alarm tendency and improved transition selectivity indicated by the summary metrics. Because the alarm labels are used only to evaluate transition-sensitive indication, these confusion matrices should be interpreted as window-level monitoring summaries rather than event-level fault-classification results.

The latency comparison in Figure 6c further supports the use of the CNN–LSTM AE as the representative autoencoder alarm-branch candidate. The LSTM AE exhibits the longest response delay, while the 1D-CNN AE shortens the latency by detecting local waveform changes more rapidly. The CNN–LSTM AE provides the shortest latency among the three autoencoder models, suggesting that the hybrid structure can respond more promptly to boundary-transition-induced waveform departures.

At the same time, the absolute F1-score and recall of the autoencoder models remain limited. The model comparison, therefore, supports the CNN–LSTM AE as a reasonable raw-window alarm-branch candidate for highlighting transition-sensitive intervals in this single-channel FBG sensing framework; however, the result should be interpreted as task-specific evidence rather than general algorithmic superiority. The alarm output remains dependent on the threshold setting, window overlap, operating condition, and the desired balance between sensitivity and conservatism.

## 5. Conclusions

This study presented a grating-based fiber-optic sensing framework for relative motor vibration-state transition monitoring under angle-dependent boundary conditions. A packaged FBG sensing unit was mounted on the motor–support structure, and the measured Bragg-wavelength sequence was interpreted as a coupled motor–support optical response. This formulation emphasizes that a single optical sensing channel installed away from the rotating shaft can still provide useful relative information about boundary-dependent motor–support vibration behavior within the tested sensing configuration.

The proposed analysis combined two complementary branches. The AVMD branch provided an interpretable time-varying narrowband spectral-response trajectory, allowing fixed-boundary behavior and dynamic boundary-transition behavior to be visualized as relative vibration-state patterns. In parallel, the raw-window autoencoder branch transformed waveform departures from learned nominal fixed-angle behavior into alarm-candidate indications. The fixed-angle observations provided stable reference responses under controlled boundary states, while the dynamic transition records showed that localized changes in the optical waveform and latent representation can be highlighted without handcrafted features, external speed references, or labeled fault data.

The comparison among LSTM AE, 1D-CNN AE, and CNN–LSTM AE showed that the CNN–LSTM configuration provided the most selective response among the tested autoencoder branches, with the highest precision, the lowest false-alarm rate, and the shortest autoencoder latency. The baseline comparison further showed that the RMS threshold alarm provided higher transition sensitivity and shorter latency, but at the cost of a higher false-alarm rate. This result indicates that the dominant transition response in the present configuration contains an amplitude-sensitive component. However, the higher false-alarm rate of the RMS threshold baseline also suggests that amplitude-only monitoring may be less selective under nominal background variations. Therefore, the CNN–LSTM AE should be interpreted as a complementary selectivity-oriented alarm branch rather than a generally superior replacement for RMS-based monitoring.

The present results support the feasibility of using a single packaged FBG channel for remote observation of relative motor vibration-state transitions under controlled boundary changes. The alarm output should be understood as a transition-sensitive indication of vibration-state departure within the tested single-channel sensing configuration. The current validation was limited to one motor platform, selected inclination conditions, one nominal drive setting, a limited set of dynamic boundary-transition records, and the tested packaged FBG sensing configuration. Future work will include repeated trials, independent speed and acceleration references, broader loading and drive conditions, start–stop transient validation as a separate task, multi-FBG spatial sensing, sensor remounting tests, temperature-drift evaluation, and field tests on different machinery. These extensions will help determine how the proposed framework can be generalized from controlled laboratory observation toward broader industrial condition-monitoring applications.

## Figures and Tables

**Table 1:** Quantitative comparison of the three autoencoder alarm-branch candidates shown in Figure 6 for the dynamic angle-transition evaluation.

Method	Precision	F1-Score	Recall	False-Alarm Rate	Latency (s)
LSTM AE	0.261	0.049	0.027	0.025	14.18
1D-CNN AE	0.486	0.138	0.080	0.028	8.97
CNN–LSTM AE	0.714	0.226	0.134	0.017	3.99

## Data Availability

The data presented in this study are available on request from the corresponding author.
